# Neurophysiological correlates of holistic face processing in adolescents with and without autism spectrum disorder

**DOI:** 10.1186/s11689-018-9244-y

**Published:** 2018-08-30

**Authors:** Sandra Naumann, Ulrike Senftleben, Megha Santhosh, James McPartland, Sara Jane Webb

**Affiliations:** 1 Berlin School of Mind and Brain, Humboldt-Universität zu Berlin, Berlin, Germany; 2 Department of Psychology, Technische Universität Dresden, Dresden, Germany; 3 Seattle Children’s Research Institute, Seattle, USA; 4 Yale University, New Haven, USA; 5 University of Washington, Washington D.C., USA

**Keywords:** ASD, Gamma-band activity, Holistic face processing, P1, N170

## Abstract

**Background:**

Face processing has been found to be impaired in autism spectrum disorders (ASD). One hypothesis is that individuals with ASD engage in piecemeal compared to holistic face processing strategies. To investigate the role of possible impairments in holistic face processing in individuals with autism, the current study investigated behavioral and electroencephalography (EEG) correlates of face processing (P1/N170 and gamma-band activity) in adolescents with ASD and sex-, age-, and IQ-matched neurotypical controls.

**Methods:**

Participants were presented with upright and inverted Mooney stimuli; black and white low information faces that are only perceived as faces when processed holistically. Participants indicated behaviorally the detection of a face. EEG was collected time-locked to the presentation of the stimuli.

**Results:**

Adolescents with ASD perceived Mooney stimuli as faces suggesting ability to use holistic processing but displayed a lower face detection rate and slower response times. ERP components suggest slowed temporal processing of Mooney stimuli in the ASD compared to control group for P1 latency but no differences between groups for P1 amplitude and at the N170. Increases in gamma-band activity was similar during the perception of the Mooney images by group, but the ASD group showed prolonged temporal elevation in activity.

**Conclusion:**

Overall, our results suggest that adolescents with ASD were able to utilize holistic processing to perceive a face within the Mooney stimuli. Delays in early processing, marked by the P1, and elongated elevation in gamma activity indicate that the neural systems supporting holistic processing are slightly altered suggesting a less automatic and less efficient facial processing system.

**Trial registration:**

Non-applicable.

**Electronic supplementary material:**

The online version of this article (10.1186/s11689-018-9244-y) contains supplementary material, which is available to authorized users.

## Background

The processing of social information in faces is crucial to communicate effectively with others [1, 2]. Faces possess two types of configural information: first-order information (repeated in every face; e.g., two eyes, above a nose, above a mouth) to enable early face detection [3, 4], and emerging second-order properties (variations in spacing between the features) to extract inter-face variance and to discriminate between faces [3, 5–7]. In configural processing, a face is therefore perceived from lower features to emergent features. In contrast, holistic processing assumes that faces are perceived immediately as undifferentiated wholes without going from first-to second-order features [7]. Configural and holistic processing have been assumed to play parallel roles within face processing [8].

Further interest in face processing is fueled by neurodevelopmental conditions such as autism spectrum disorders (ASDs), which are characterized by early and pervasive social communication and interaction impairments [9]. Individuals with ASD show an enhanced reliance on, or a greater scanning of, unusual face parts (i.e., mouth instead of eyes) [10–12]. The integration of visual information into a meaningful whole may be impeded by processing predominantly first- rather than second-order features leading to a part-based processing style [13–15].

Face inversion paradigms have been used to examine holistic and configural processing in ASD (e.g., [15]). The inversion of a human face may disrupt configural processing [16, 17]. The extraction of first-order information remains intact regardless of stimulus’ orientation [3]. In face inversion tasks, accuracy rates for upright faces compared to inverted faces were higher for neurotypical controls [5]. For upright stimuli, holistic and configural strategies may work together, which contributes to higher accuracy rates, whereas a stronger reliance on first-order features is necessary for inverted faces, which contributes to lower accuracy levels. In contrast, individuals with ASD displayed similar detection rates for upright and inverted faces [18]. This pattern of results supports the idea of a part-based processing strategy in which individuals with ASD predominantly rely on first-order information for upright and inverted face stimuli. Reaction time analyses of face inversion paradigms complement these findings as controls are faster in making their decisions compared to individuals with ASD (e.g., [19]). There have been, however, contrasting results which demonstrate similar face detecting rates in both groups [15, 20, 21] or even better performance in the ASD group compared to controls [20]. In fact, a recent systematic review suggested an intact face inversion effect for the ASD group in most studies [22].

Individuals with ASD may engage in similar face processing strategies as controls [12], but due to a lack of attention to faces from an early age [2], individuals with ASD may develop less expertise in face identification and discrimination [23]. Similar face detection rates for controls and the ASD group were also observed when cueing to relevant parts of the face [10]. Researchers have therefore suggested a quantitative instead of a qualitative difference of face perception in ASD [22, 24].

The disentanglement of holistic and configural processes is another challenge of face inversion tasks [7]. To address this, studies employ the Mooney face task to specifically trigger holistic processes [16, 19, 25, 26]. Mooney stimuli give rise to faces by the two-tone composition of black and white parts [16]. Extensive binding and holistic processes are required to perceive them as faces because they contain few explicit local features [25, 27, 28]. Upright presented Mooney stimuli are thought to recruit more efficient holistic processes, whereas inverted Mooney stimuli severely hinder face abstraction [8, 29]. As before, some studies reported a face inversion effect with Mooney stimuli for the ASD group (e.g., [20]), while others failed to find it (e.g., [19]).

Part of the discrepancy in these result patterns may be related to the inclusion of individuals spanning broad age bands. Holistic face processing was suggested to be impaired in children with ASD (aged 8 to 13 years) who displayed lower accuracy for inverted compared to upright face stimuli [21] and showed less sensitivity to configuration of the faces potentially due to holistic processing deficit or a lack of expertise with faces [30]. McPartland et al. (2004) demonstrated ERP differences in basic face vs. house comparisons in adolescents and adults 15 to 42 years; however, Webb et al. (2012) in adults 18-to-44-year-olds with ASD did not find altered face vs. house ERP activity but did find differences between groups in face inversion processing [23]. In a sample of 9-to 45-year-olds with ASD, O’Connor et al. (2005) found that the younger group with ASD (9 to 15 years) displayed no difference in task performance (compared to the controls), whereas adults with ASD (18 to 45 years) showed deficits across all emotion categories, which the authors suggested reflected indicating a general facial configuration deficit for adults with ASD [17]. One possible source of confound in these papers is the inclusion of the transitional stage of adolescence within either the child or adult groups. It may be of importance to examine manifestations and trajectories of face processing differences separately for adolescents, particularly as orientation processing and some aspects of holistic processing may become mature in childhood (e.g., [31]), but other neural markers of face sensitivity do not become mature until late-adolescence [32].

General face processing differences in adolescents with and without ASD may be represented by altered patterns at the neural level, specifically in the ERP components P1 and N170 which reflect attentional and perceptual aspects of the neural circuitry of face perception [1, 12, 33, 34]. Of importance, based on the latency of these components, this neural activity often precedes behavioral responses about face stimuli. The P1 event-related potential (ERP) component is a positive deflection around 100 ms associated with visual attention [35, 36]. In children and young adolescents with ASD compared to controls, Hileman et al. (2011) found smaller P1 amplitudes (but not latency) for inverted compared to upright faces while Neuhaus et al. (2016) found an inversion effect in the control group for latency (but not amplitude) which was not apparent in children and adolescents with ASD [37]. Within an adult sample, differences in P1 amplitude (but not latency) for inversion across groups were reported [38].

The N170 component reflects face categorical processing (relative to other objects), as well as eye featural sensitivity [39, 40]. In 3- to 4- and 3- to 6-year-old children with ASD, N170 latencies were longer and amplitudes smaller compared to controls in face vs. object perception tasks [2, 41]. Studies of early and late adolescence in ASD displayed a similar pattern of delayed N170 latencies without differences in N70 amplitudes to faces compared to controls [42], but this was not found in another report [37]. It is possible, that the inconsistent finding of a face inversion effect in behavior and ERPs is also associated with the underlying developmental trajectory of holistic processing and with different stimulus types and comparisons altering the extent to which the sources contributing to the P1 or N170 are implicated.

Additional EEG signal properties may inform our understanding of the mechanisms of holistic processing. The rhythmic synchronization of neural discharges in the gamma-band (> 25 Hz) relates to the ‘binding problem’ that is, the question of how various visual features are integrated to a coherent object representation [26, 43, 44]. It is associated with the pyramidal network’s synchronization of excitatory and inhibitory interneurons [43]. Gamma-band activity (GBA) has also been connected to working memory and visual attention processes [43, 45]. The match-and-utilization model (MUM) predicts that meaningful objects such as upright faces lead to stronger GBA compared to inverted faces [46]. GBA in the lower range (25–45 Hz, 150–250 ms) has been shown to be sensitive to inversion of faces with lower activity for inverted compared to upright faces [39] and greater for faces compared to scrambled faces in neurotypical controls around 200 ms [4, 47]. Adults with ASD displayed lower levels of GBA in the lower gamma-band over occipital areas within a passive face viewing task with peak differences between 250 and 450 ms [45] or during a Mooney face inversion task between 100 and 300 ms [19]. GBA of adults with ASD was not sensitive to inversion of face in the lower gamma-band range at frontal sites, whereas controls showed a larger burst for upright faces [48]. These abnormalities in GBA may underlie disruptions in face processing in ASD at a very basic level [45].

Taken together, there are documented differences in face processing in behavioral and neural activity in children and adults with ASD but less is known about holistic face processing during adolescence as most studies have included adolescents either with younger or adult participants rather than as a targeted group. This may be an age period of particular importance as the P1 and N170 (amplitude, latency, and response characteristics) as a marker of early stage face processing becomes adult-like in the quality of the response pattern but still quantitatively differs in amplitude and latency [32]. Therefore, we aimed at investigating behavioral (detection rate/response times) and neurophysiological correlates (P1/N170 component/gamma-band activity) of holistic face processing in a narrow range sample of adolescents with ASD and sex-, age-, and IQ-matched neurotypical controls. EEG was collected while adolescents completed an inversion task with Mooney stimuli.

If adolescents with neurotypical development show effective holistic processes, and in contrast, holistic processing is impaired in the ASD group, then we predict that (1) slower response times as well as reduced face detection rates would occur in the ASD group compared to controls, suggestive of reduced holistic face perception and stronger focus on first-order features. (2) P1 latency would not be modulated by Mooney stimuli detected as faces, whereas P1/N170 amplitudes and N170 latencies to Mooney stimuli detected as faces would be slower and of less amplitude in ASD compared to controls. (3) Controls but not the ASD group would display greater P1/N170 amplitude and faster N170 latency to stimuli perceived as face compared to non-face responses. (4) Gamma power in the lower gamma-band range (25–45 Hz; associated with perceptual binding) would be smaller in the ASD compared to the control group in early and late time windows for anterior and posterior clusters.

## Methods

### Participants

The local Institutional Review Board approved the protocol, all adolescents provided written assent, and a parent provided written consent for participation. Adolescents with ASD met research diagnostic criteria based on the Autism Diagnostic Observation Schedule (ADOS) [49], criteria on the social and communication domains of the Autism Diagnostic Interview-Revised (ADI-R) [50], and DSM-IV criteria based on expert clinical diagnostic judgment [51]. Adolescents with typical development had no history of developmental delay or concerns about autism-related behaviors. Exclusionary criteria for adolescents with ASD and controls included performance IQ scores < 80 (Wechsler Intelligence Scale III; WISC), known genetic disorders, seizures, significant sensory or motor impairment, major physical abnormalities, serious head injury, and use of anticonvulsant or barbiturate medications. Performance IQ was employed as criteria because the tasks across the full protocol focused on non-verbal visual processing. Additional exclusionary criteria for controls included birth or developmental abnormalities, psychotropic medication usage, and a first-degree relative with ASD. Sixty-eight adolescents were enrolled in the study. Participants were matched based on their age and sex followed by bin-matching with regard to their performance IQ during the screening session. Thirty participants were excluded from the final analysis: 8 participants were disqualified after enrollment (non-compliance or too low IQ), 8 datasets had EEG file errors that resulted in unusable data, 6 had significant EEG artifacts (e.g., excessive movement), and 8 did not show visible ERP components after averaging. The final sample consisted of 19 controls and 19 participants with ASD. No group differences for age, gender, or performance IQ were detected. There were no significant differences in characteristics between those that were included in the analysis and those that were not (*p*s > .05). Demographic characteristics are provided in Table 1.

**Table 1:** Means and standard deviations for gender, age, and IQ scores of controls and the ASD group

	Controls (*N* = 19)		ASD group (*n* = 19)		*χ* ^*2*^*/t* value	*p* value
	Mean	SD	Mean	SD		
Gender (M:F)	16:3		16:3		*χ* ^*2*^(1) = 0.000	1.000
Age (years)	13.950	1.268	14.000	1.667	*t*(36) = 0.110	.913
P IQ	112.790	16.755	115.737	14.681	*t*(36) = 0.577	.568
FS IQ	113.630	17.150	109.370	13.039	*t*(36) = − 0.863	.394

Note. *ASD* = autism spectrum disorder, *P IQ* = Wechsler Intelligence Scale III Performance IQ, *FS IQ* = Wechsler Intelligence Scale III Full Performance, *SD* = standard deviation

### Apparatus and stimuli

**Fig. 1:**
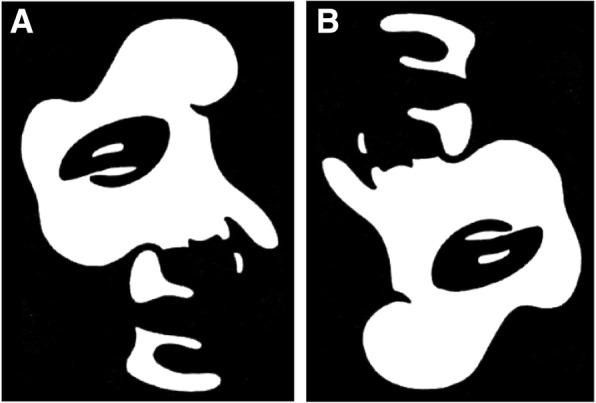
Examples of upright (**a**) and inverted (**b**) Mooney face stimuli

The current study used a set of 50 Mooney face stimuli (5.9° by 7.9°), which are degraded, 2-tone pictures of human faces [52] (see Fig. 1, Mooney face stimulus examples). They were presented upright and inverted to manipulate holistic face perception.

### Procedure

Adolescents completed a training block, consisting of four trials in which Mooney stimuli were either presented upright (*n* = 2) or inverted (*n* = 2). They were asked to indicate whether they perceived a face or not. During the training trials, the goal was to practice mapping the right/left button press to the decision of face/no face. After the mapping was understood, adolescents started with the actual task in which they saw a random sequence of upright and inverted Mooney stimuli. Participants were asked to answer as spontaneously and quickly as possible. Face and no face button position was balanced across participants.

The experiment consisted of 200 trials, presented in four 50 image blocks. A break of participant-determined length separated each block. In each trial, a gray background was presented for 500 ms (baseline) followed by a Mooney stimulus for 500 ms displayed on a gray background. The inter-trial interval (ITI) varied between 2000 and 2500 ms. Adolescents could indicate their decision across the entire stimulus presentation and ITI.

#### Electrophysiological recordings

EEG was recorded with a 128-channel Geodesic Sensor NetAmps 200 in Net Station 2.0 (Electric Geodesic, Inc. Eugene OR), with a sampling rate of 500 Hz, and experimental control through E-Prime 1.0 software. In a dimly-lit, sound-attenuated room, adolescents sat approximately 24 inches from the stimulus monitor and used buttons 1 (left most button, left index finger) and 5 (right most button, right index finger) on a 5-button box for experimental response.

#### Processing

All procedures were conducted with MATLAB’s Toolbox EEGLAB (The MathWorks, Natick, MA). Re-sampling of the data to 250 Hz and filtering (0.1 Hz highpass; 100 Hz lowpass; 60 Hz notch) preceded the exclusion of bad channels (impedances over 200 KOhm, drifting channels). Data was re-referenced to average reference, segmented into epochs (− 500 to 1000 ms) for each condition and baseline-corrected to 500 ms pre-stimulus interval. Hand editing was done as a first artifact rejection step to address “non-stereotyped” noise (e.g., pulling the cap) prior to conducting an independent component analysis (ICA). With the help of the EEGLAB plugin SASICA [53], components such as those containing electrical noise, ocular, or head movements were identified. Visual inspection served as final judgment on rejecting bad components. Lastly, excluded channels were interpolated using spherical interpolation.

#### ERP data

Based on a study of Webb et al. (2012), amplitudes for the P1 and N170 component in adolescents were chosen from a posterior medial left cluster (electrodes 65, 70, 71, and 75) and a posterior medial right cluster (electrodes 83, 84, 90, and 91; also see Additional file 1). The first positive peak was defined as the P1 component and the N170 component was specified as the first negative deflection following the P1. Temporal windows for extracting the ERP components were visually inspected for developmental shifts in latency, amplitude, and morphology [32, 42]. Overall time windows ranged from 70 to 170 ms (P1) and 120 to 220 ms (N170). Amplitudes and latencies were extracted across the selected clusters within the designated time windows separately for the clusters of the left and right hemisphere for the P1 and N170 component. P1 and N170 components had to be present in 50% of the defined electrode cluster to be further included. Data was separately inspected for upright presented stimuli (trials face response: *M* = 65.210, SD = 11.928; trials no face response: *M* = 17.820, SD = 9.320) and inverted presented stimuli (trials face response: *M* = 44.000, SD = 20.254; trials no faces response: *M* = 37.530, SD = 15.446). At least 20 trials in a condition were necessary to be included in further analyses. Instead of peak amplitude, mean P1 amplitude was calculated to account for the noise level of the waveform [54]. To account for influences of the preceding P1, adjusted N170 amplitudes and latencies were calculated by subtracting the P1 peak amplitude from the N170 peak amplitude, and the P1 peak latency from the N170 peak latency [55]. Lastly, grand average waveforms were calculated for both groups.

#### Time-frequency-analysis

Gamma-band power (25–45 Hz) was calculated in 50 linear steps using complex Morlet wavelets (c.f. [56]). The wavelets were defined as ${\left({\sigma}_t\sqrt{\pi}\right)}^{-\frac{1}{2}}\exp \left(-\frac{t^2}{2{\sigma}_f^2}\right)\exp \left(2 i\pi {f}_0t\right)$*,* with *σ*_*f*_ = 1/2πσ_*t*_*,* where *t* is time, *f*_*0*_ is frequency, and where *σ*_*f*_ and *σ*_*t*_ denote the length of the wavelet in the frequency and time domain. The ratio *f*_*0*_*/σ*_*f*_ was set to 5. We focused on induced gamma (i.e., non phase-locked gamma power) by obtaining time-frequency transforms of single epochs first and then averaging them across trials for each condition (c.f. [57, 58]). The time-frequency data was normalized to baseline (− 350 to − 50 ms) by applying a *Z*-transform, where the difference between signal and baseline was divided by the standard deviation of the baseline according to formula (1):

$$ {Z}_{tf}=\frac{{\mathrm{activity}}_{tf}-\overline{{\mathrm{baseline}}_{tf}}}{\sqrt{n^{-1}\sum \limits_{i=1}^n{\left({\mathrm{baseline}}_{tf}-\overline{{\mathrm{baseline}}_{tf}}\ \right)}^2}} $$

in which *Z* denotes *Z* value, *t* denotes time, *f* frequency, and *n* denotes the number of time points in the baseline. *Z* values from the electrode clusters of interest included the P1/N170 posterior inferior left and right clusters. Based on visual inspection of the scalp map distribution, an anterior left and anterior right cluster was added (left cluster electrodes 19, 23, 24, and 27; right cluster electrodes 2, 3, 9, and 10). Further, based on the time-frequency plots, two time windows were identified for analysis (50–200 ms; 200–350). Signal was averaged separately for clusters across the 25–45 Hz band and for each time window. These values were then averaged across participants for each group.

### Statistical analysis

After processing, too few participants had data available for upright Mooney faces not detected as faces. This condition was therefore not included. Thus, we examined the contrasts of face responses to upright and inverted stimuli and face “no face” responses for inverted stimuli within the ERP and gamma analyses.

For face detection rates and reaction times, trials were averaged based on stimulus’ orientation (upright/inverted) for stimuli detected as faces. They were submitted to repeated-measures analyses of variances (ANOVA) with orientation (upright/inverted) as within-factor and group (ASD group/controls) as between-factor.

To contrast ERP responses for face responses to upright and inverted stimuli, mean P1 amplitudes and latencies and adjusted N170 amplitudes and latencies were entered into separate repeated-measures ANOVA including the factors orientation (upright/inverted) and hemisphere (left/right) as within-factors and group (ASD group/controls) as between-factor.

To compare face to no face responses, mean P1 amplitudes and latencies and adjusted N170 amplitudes and latencies were averaged for these categories within the inverted condition. Afterwards, values were submitted to separate repeated-measures ANOVA with percept (face/no face) and hemisphere (left/right) as within-factors and group (ASD group/controls) as between-factor.

To compare GBA responses, a repeated-measures ANOVA with the within factors percept (face/no face), time window (50–200 ms/ 200–350 ms), cluster (anterior/posterior), and hemisphere (left/right) as well as the between factor group (ASD group/controls) was calculated.

All statistical analyses were performed with the IBM (Armank, NY) SPSS Statistics 14.0 software package and MATLAB (The MathWorks, Natick, MA). All analyses were followed up with inclusion of FS IQ or age as a covariate; these covariates did not change the results and findings are reported without the covariates. Significant main effects and interactions were followed by subsequent 1-way ANOVAs for the groups or by post hoc Bonferroni-corrected contrasts. For all analyses, the significance level was set at α < 0.05.

## Results

### Behavioral performance

#### Detection rate

**Fig. 2:**
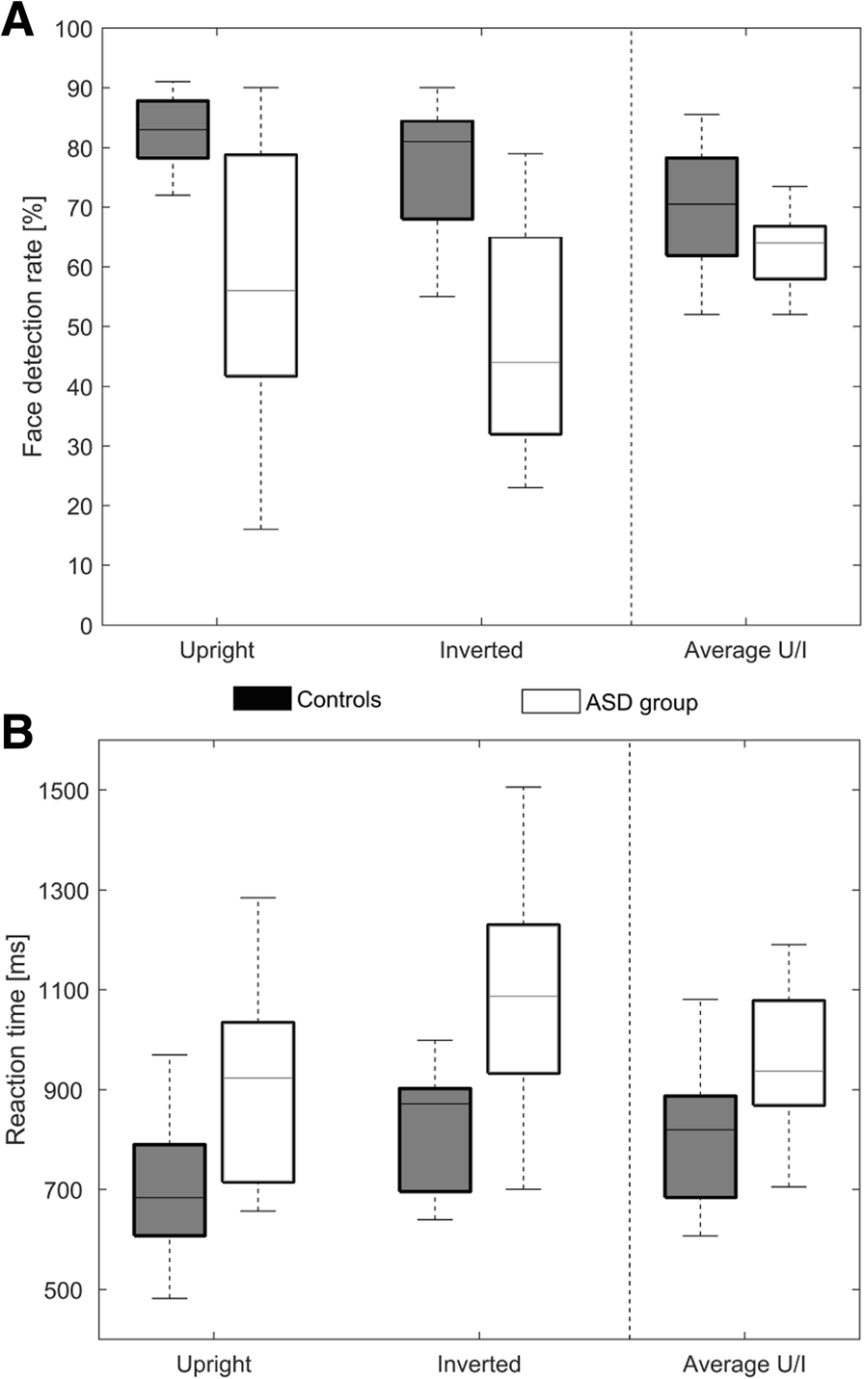
Behavioral performance differences between controls and participants with ASD. Face detection rate in percent (**a**) and response time in milliseconds (**b**) for face decisions to upright and inverted Mooney stimuli for controls (black boxplots) and participants with ASD (white boxplots)

As hypothesized, Controls detected significantly more Mooney stimuli as faces compared to participants with ASD (*F*(1, 36) = 6.272, *p* < .05, η_p_^2^ = .148). Both groups identified more Mooney stimuli as faces in the upright compared to the inverted presentation (*F*(1, 36) =37.316, *p* < .001, η_p_^2^ = .982). There was no orientation ***×*** group interaction (*F*(1, 36) = 0.089, *p* = .768, η_p_^2^ = .148) (Fig. 2).

#### Response time

In line with our hypothesis, the control group was faster than the ASD group to detect a face for upright and inverted Mooney stimuli (*F*(1, 36) = 6.106, *p* < .05, η_p_^2^ = .145). Both groups were faster to detect an upright Mooney stimulus as opposed to an inverted Mooney stimulus as a face (*F*(1, 36) = 92.506, *p* < .001, η_p_^2^ = .720). There was no group x orientation interaction (*F*(1, 36) = 0.159, *p* = .692, η_p_^2^ = .004) (Table 2).

**Table 2:** Means and standard deviations for face detection rate and reaction times for controls and the participants with ASD

	Controls (*n* = 19)		ASD group (*n* = 19)	
	Mean	SD	Mean	SD
Hits upright stimuli (hus) (%)	81.250	8.097	75.474	12.607
Hits inverted inverted (his) (%)	56.200	21.279	47.737	18.624
Reaction time hus (ms)	829.979	196.972	952.684	221.415
Reaction time his (ms)	1049.970	226.513	1188.531	221.813

Note. *ASD* = autism spectrum disorder, *SD* = standard deviation

### ERP analysis for face decisions in upright vs. inverted stimuli

#### P1 latency

In contrast to our hypothesis, a between-group comparison revealed a significant effect for group (*F*(1, 36) = 5.692, *p* < .05, η_p_^2^ = .137) with controls compared to the ASD group displaying faster P1 latencies for trials with a face decision. There was no effect of orientation (*F*(1, 36) = 0.907, *p* = .347, η_p_^2^ = .025) or interaction of group ***×*** orientation (*F*(1, 36) = 0.004, *p* = .950, η_p_^2^ = .000). When averaged across group and orientation, no hemisphere differences were detected (*F*(1, 36) = 2.899, *p* = .097, η_p_^2^ = .075), nor interactions with group (*F*(1, 36) = 0.450, *p* = .507, η_p_^2^ = .012) or orientation (*F*(1, 36) = 0.529, *p* = .472, η_p_^2^ = .014). The significant 3-way interaction of group ***×*** orientation ***×*** hemisphere (*F*(1, 36) = 9.339, *p* < .05, η_p_^2^ = .206) led to subsequent 1-way ANOVAs separated for group. None of the contrasts (separated by group) displayed significant outcomes.

#### P1 amplitude

Contrary to expectations, the ASD group showed similar P1 amplitudes compared to controls when Mooney stimuli were detected as faces (*F*(1, 36) = 1.068, *p* = .308, η_p_^2^ = .029). There was no main effect of orientation (*F*(1, 36) = 1.013, *p* = .321, η_p_^2^ = .027), nor interaction with group (*F*(1, 36) = 3.014, *p* = .091, η_p_^2^ = .077). P1 amplitudes did not differ across hemisphere (*F*(1, 36) = 0.944, *p* = .338, η_p_^2^ = .026). No significant interactions of hemisphere ***×*** group (*F*(1, 36) = 0.243, *p* = .625, η_p_^2^ = .007), hemisphere ***×*** orientation (*F*(1, 36) = 0.000, *p* = .998, η_p_^2^ = .000), nor hemisphere ***×*** group ***×*** orientation (*F*(1, 36) = 1.337, *p* = .255, η_p_^2^ = .036) were observed.

#### N170 latency

Faster latencies were expected for controls compared to the ASD group when detecting a face. In contrast to our hypothesis, the ASD group showed similar N170 latencies compared to controls (*F*(1, 36) = 0.796, *p* = .378, η_p_^2^ = .022), not influenced by orientation (*F*(1, 36) = 2.191, *p* = .147, η_p_^2^ = .057). No group ***×*** orientation interaction (*F*(1, 36) = 0.280, *p* = .600, η_p_^2^ = .008) or differences between hemispheres (*F*(1, 36) = 2.338, *p* = .245, η_p_^2^ = .037) were detected. Hemisphere did not interact with group (*F*(1, 36) = 1.396, *p* = .245, η_p_^2^ = .037) or orientation (*F*(1, 36) = 0.121, *p* = .730, η_p_^2^ = .335). The 3-way interaction of group ***×*** orientation ***×*** hemisphere was not significant (*F*(1, 36) = 3.673, *p* = .063, η_p_^2^ = .093).

#### N170 amplitude

Contrary to expectations, controls and participants with ASD showed similar N170 amplitudes when detecting faces within the Mooney stimuli (*F*(1, 36) = 0.492, *p* = .488, η_p_^2^ = .013). Orientation did not influence N170 amplitudes (*F*(1, 36) = 0.393, *p* = .535, η_p_^2^ = .011) or interact with group (*F*(1, 36) = 0.780, *p* = .383, η_p_^2^ = .021). A significant difference between hemispheres (*F*(1, 36) = 18.135, *p* < .001, η_p_^2^ = .335) indicated larger N170 amplitudes in the right compared to the left cluster (*p* < .001). There was no interaction of hemisphere ***×*** group (*F*(1, 36) = 2.031, *p* = .163, η_p_^2^ = .053), hemisphere ***×*** orientation (*F*(1, 36) = 0.001, *p* = .979, η_p_^2^ = .000), nor hemisphere ***×*** orientation ***×*** group (*F*(1, 36) = 0.155, *p* = .696, η_p_^2^ = .004).

### ERP analysis for face vs. no face decisions in inverted Mooney stimuli

#### P1 latency

**Fig. 3:**
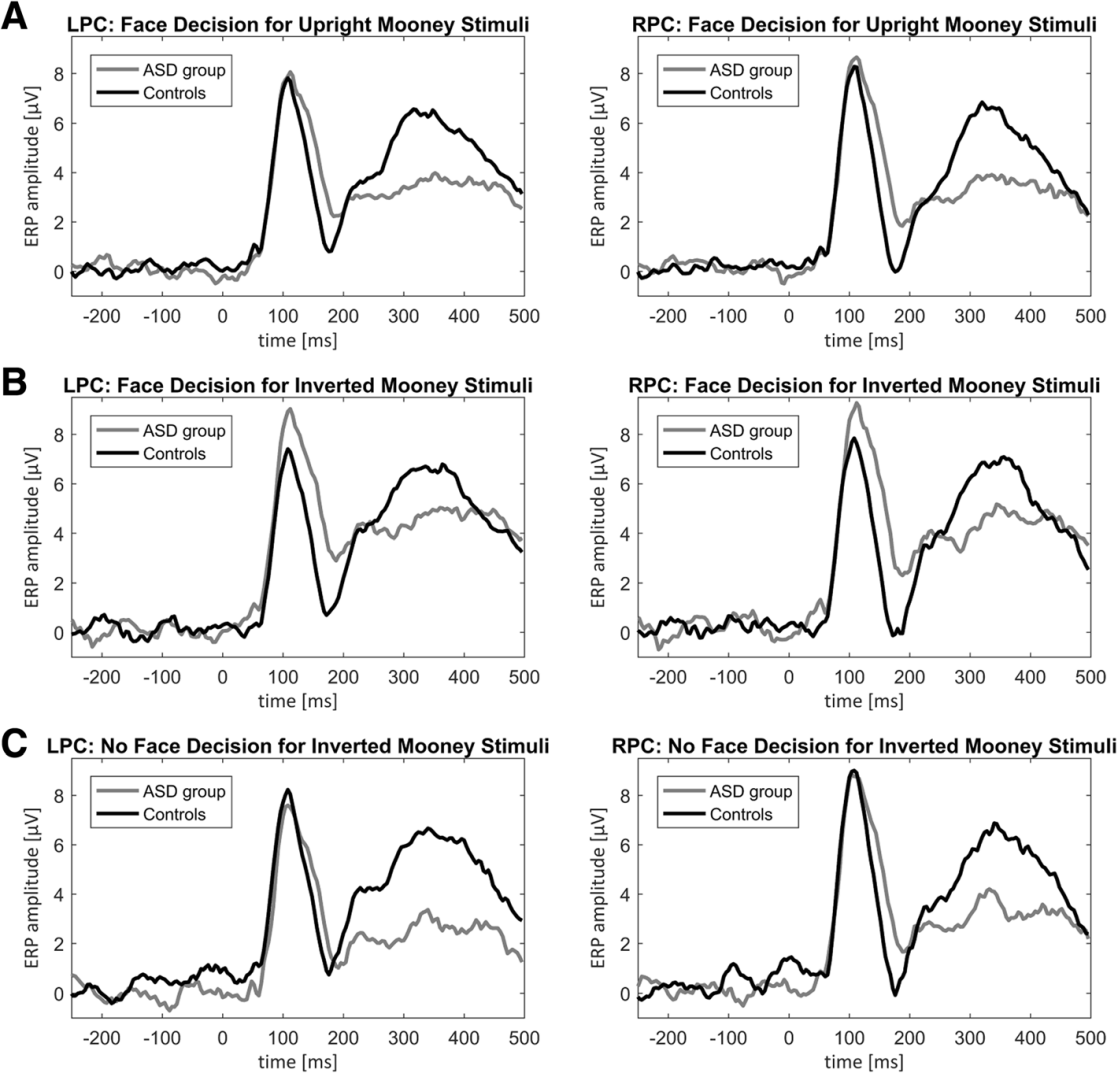
ERP differences for face and no face decisions between groups. ERP plots are represented for face decisions in upright Mooney stimuli (**a**), face decisions in inverted Mooney stimuli (**b**), and no face decisions in inverted Mooney stimuli (**c**) separately for the left posterior cluster (LPC) and right posterior cluster (RPC) for controls (gray line) and the ASD group (black line)

Contrary to expectations, a main effect of group (*F*(1, 36) = 5.349, *p* < .05, η_p_^2^ = .129) indicated longer latencies for the ASD group compared to controls across conditions. Latencies were not modulated by percept (*F*(1, 36) = 0.704, *p* = 407, η_p_^2^ = .019) or a percept ***×*** group interaction (*F*(1, 36) = 0.028, *p* = .868, η_p_^2^ = .001), indicating that the latency difference was not due to face detection differences. The effect of hemisphere was not significant (*F*(1, 36) = 3.249, *p* = .080, η_p_^2^ = .083), nor did hemisphere interact with percept (*F*(1, 36) = 0.088, *p* = .769, η_p_^2^ = .002), group (*F*(1, 36) = 0.767, *p* = .387, η_p_^2^ = .021) or display 3-way interaction (*F*(1, 36) = 2.818, *p* = .102, η_p_^2^ = .073) (Fig. 3).

#### P1 amplitude

In contrast to our hypothesis, there was no significant main effect of group suggesting that P1 amplitudes for face vs. no face decision did not differ between controls and the ASD group (*F*(1, 36) = 0.558, *p* = .460, η_p_^2^ = .015). Percept did not yield a significant effect (*F*(1, 36) = 0.105, *p* = .748, η_p_^2^ = .003). A significant percept ***×*** group interaction was observed (*F*(1, 36) = 5.699, *p* < .05, η_p_^2^ = .137); however, post hoc analyses showed no difference of percept for controls (*p* = .153) or the ASD group (*p* = .063). The effect of hemisphere was significant (*F*(1, 36) = 7.953, *p* < .05, η_p_^2^ = .181). Amplitudes in the right cluster were larger compared to the left cluster (*p* > .05). Hemisphere did not interact with group (*F*(1, 36) = 0.026, *p* = .872, η_p_^2^ = .001), nor percept (*F*(1, 36) = 0.977, *p* = .330, η_p_^2^ = .026), nor a 3-way interaction (*F*(1, 36) = 1.346, *p* = .254, η_p_^2^ = .036).

#### N170 latency

We expected similar N170 latencies for face and no face decisions for the ASD group which were hypothesized to be delayed compared to controls. In contrast to our hypothesis, controls and participants with ASD displayed similar N170 latencies (*F*(1, 36) = 0.155, *p* = .696, η_p_^2^ = .004). N170 latencies were not modulated by percept (*F*(1, 36) = 1.670, *p* = .204, η_p_^2^ = .044) or percept ***×*** group interaction (*F*(1, 36) = 1.670, *p* = .204, η_p_^2^ = .044). None of the factors (hemisphere: *F*(1, 36) = 1.604, *p* = .213, η_p_^2^ = .043; hemisphere ***×*** group: *F*(1, 36) = 1.313, *p* = .259, η_p_^2^ = .035; hemisphere ***×*** percept: *F*(1, 36) = 0.018, *p* = .893, η_p_^2^ = .001, hemisphere ***×*** percept ***×*** group: *F*(1, 36) = 1.495, *p* = .229, η_p_^2^ = .040) reached significance.

#### N170 amplitude

Contrary to expectations, controls showed similar N170 amplitudes compared to the ASD group (*F*(1, 36) = 0.922, *p* = .343, η_p_^2^ = .025). There was no effect of percept (*F*(1, 36) = 0.019, *p* = .892, η_p_^2^ = .001), nor did percept interact with group (*F*(1, 36) = 1.049, *p* = .313, η_p_^2^ = .028). A significant effect for hemisphere (*F*(1, 36) = 20.744, *p* < .001, η_p_^2^ = .366) was observed, indicating that larger N170 values were found within the right cluster (*p* < .001). There was no interaction of hemisphere ***×*** group (*F*(1, 36) = 0.250, *p* = .620, η_p_^2^ = .007), hemisphere ***×*** percept (*F*(1, 36) = 0.386, *p* = .539, η_p_^2^ = .011) or hemisphere ***×*** percept ***×*** group (*F*(1, 36) = 0.566, *p* = .121, η_p_^2^ = .065).

### Summary ERP analysis

To summarize our ERP results, controls and individuals with ASD showed similar P1 and N170 morphologies. They only differed with regard to their P1 latencies. Controls displayed faster latencies than the ASD group for face decisions across inverted and upright Mooney stimuli and for face vs. no face decision for inverted Mooney stimuli. Across groups, N170 amplitudes were larger in the right hemisphere for face decisions. For the face vs. no face contrast in inverted Mooney stimuli, P1 amplitudes were larger in the right compared to the left cluster.

### Time frequency analyses face vs. no face decisions in inverted Mooney stimuli

We hypothesized larger GBA for controls compared to the ASD group. Contrary to expectations, groups did not differ in their general GBA (*F*(1, 36) = 0.407, *p* = .528, η_p_^2^ = .011). Whether they detected a face or not did not influence GBA levels (*F*(1, 36) = 0.049, *p* = .826, η_p_^2^ = .001), nor was there a percept × group interaction (*F*(1, 36) = 0.056, *p* = .814, η_p_^2^ = .002). GBA levels significantly differed across time (*F*(1, 36) = 7.888, *p* < .01, η_p_^2^ = .158) with larger activity in the early (50–200 ms) compared to the later time window (200–350 ms). The significant time × group interaction (*F*(1, 36) = 5.392, *p* < .05, η_p_^2^ = .110) indicates that controls showed larger GBA levels within the first time window (*p* < .001), whereas GBA levels for the ASD group were equal across time (*p* = .742). None of the effects of hemisphere reached significance (hemisphere: *F*(1, 36) = 0.393, *p* = .535, η_p_^2^ = .011; hemisphere × group: *F*(1, 36) = 0.003, *p* = .986, η_p_^2^ = .000; hemisphere × percept: *F*(1, 36) = 0.081, *p* = .778, η_p_^2^ = .002; hemisphere × time: *F*(1, 36) = 0.079, *p* = .781, η_p_^2^ = .002). GBA levels were larger for anterior compared to the posterior cluster (*F*(1, 36) = 8.799, *p* < .01, η_p_^2^ = .189). There was no significant cluster × group interaction (*F*(1, 36) = 1.690, *p* = .202, η_p_^2^ = .036), nor cluster × percept interaction (*F*(1, 36) = 0.084, *p* = .774, η_p_^2^ = .002). GBA levels of clusters did, however, differ between time windows (*F*(1, 36) = 10.389, *p* < .001, η_p_^2^ = .223). A larger reduction of activity from the early to the later time window in posterior (*p* < .001), but not within the anterior cluster (*p* = .368), was detected. Cluster did not interact with hemisphere (*F*(1, 36) = 0.005, *p* = .983, η_p_^2^ = .000). No significant 3- or 4-way interactions were observed.

#### Summary time frequency analysis

**Fig. 4:**
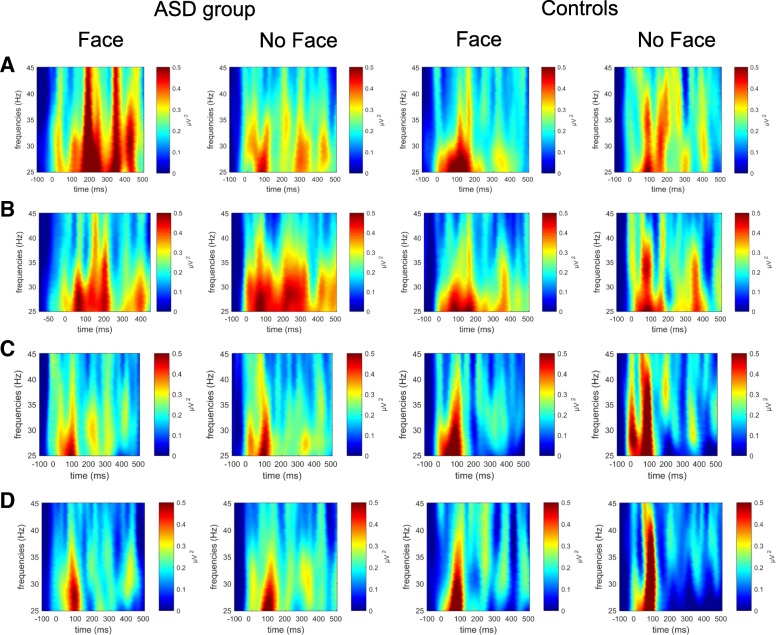
Group differences in gamma-band activity for controls and adolescents with ASD. Time-frequency plots for face and no face decisions of the ASD group and controls at the anterior left cluster (**a**), anterior right cluster (**b**), posterior left cluster (**c**), and posterior right cluster (**d**). The colored scales indicate *Z*-transformed power values

Groups did not differ in their general GBA to Mooney stimuli, regardless of orientation or percept. Controls displayed significant decreases in GBA levels in frontal clusters across time. This decrease in GBA did not occur in participants with ASD. For both groups, GBA was larger for the anterior cluster and activity showed larger decreases at the posterior compared to the anterior cluster across time (Fig. 4).

## Discussion

The present study yielded insight into holistic and configural face processing in ASD and neurotypical adolescents by examining behavioral performance (detection rate, response time) and neural correlates (P1, N170, gamma-band activity) with a Mooney stimuli inversion task. The behavioral responses suggest that mechanisms for holistic face processing are in place for both groups. Higher face detection rates for Mooney stimuli in upright orientation compared to inverted were found for both controls and the ASD group. The finding is consistent with previous studies, which also found an intact face inversion effect for individuals with ASD [15, 20–22]. While we cannot eliminate the potential that a part-based processing style influenced face detection rates in the ASD group for prior reports utilizing upright and inverted stimuli [18], our high rates of face identification are unlikely to be due to any type of parts-based system given the nature of the Mooney stimuli. Despite a similar impact of orientation on behavioral decisions of “face-ness” for both groups, adolescents with ASD were less likely to perceive Mooney stimuli as faces than controls and displayed longer reaction times to make a face decision, which is in line with another study that employed Mooney stimuli [19]. Besides intact holistic face processing, the finding also supports a quantitative instead of a qualitative face perception difference in ASD [22, 24].

The ERP results also implicate group differences in the early neural circuitry within the visual processing system thought to reflect attentional (e.g., P1), rather than perceptual (e.g., N170) processes specific to faces. Within the comparison of face responses contrasting upright and inverted Mooney stimuli, controls displayed faster P1 latencies compared to the ASD group, whereas no differences for N170 amplitude or latency could be detected between groups and by orientation. This finding is in contrast to delayed N170 latencies to realistic faces for individuals with ASD [42]. Within the contrast of face and no face responses for inverted stimuli, controls also showed faster P1 latencies compared to the ASD group. Similarly, to the first comparison, no group differences were detected for N170 amplitude or latencies, which is also in contrast to previous findings [23, 42]. Consistent with other studies utilizing facial stimuli, we did find larger N170 amplitudes in the right compared to the left cluster [4].

We investigated the lower gamma-band (25–45 Hz) to examine feature binding processes proposed to underlie deriving a face percept from the black and white Mooney images. No group differences were found in gamma-band activity for groups across clusters, which contrasts previous findings of *more* GBA in occipital areas for controls compared to adolescents with ASD [45] or *more* GBA in ASD compared to controls [19]. Both groups showed similar activity in the early window, overlapping the P1 and the start of the N170 component. Former studies found larger GBA for controls in comparison to the ASD group [19]. However, prolonged gamma-band activity for the ASD group was detected in comparison to the control group. Increased temporal activity was also found in similar time windows for adolescents with ASD [45].

General early stage processing of Mooney stimuli was identified by the P1 component activity within both comparisons. The P1 is typically associated with early visual attention [35, 36] and source-localized to the visual association cortex [59]. In our experiment, the task emphasized attention toward the stimulus to determine “face-ness,” while maintaining a 2-button response mapping. Thus, the task protocol required sustained attention and elicited a large P1 component in both groups. It is also possible that our directions provided a strategy that helped to “normalize” engagement of the face processing circuitry, as suggested by consistent morphology of the component across groups and individuals, with quantitative modulation of latency. Thus, the basic attention and processing mechanisms seem to be available in individuals with ASD and can be manipulated to produce greater responses by directing attention [12, 37]. This is in line with another study that found similar face detection rates after directing the attention to parts of the face [10].

Early stage perceptual face processing has been historically assessed by examining response patterns of the N170 component and later GBA (e.g., 150 to 250 ms or 200 to 300 ms) [4, 39]. In contrast to former studies, we did not find any differences in N170 latency or amplitude between adolescents with and without ASD [42]. Although the N170 was right-lateralized in both groups as previously reported [17, 39], it is worthy to note that most studies employ natural faces and previous research suggests that intact natural faces and eyes result in greater and faster N170 responses [60], and schematic face stimuli might induce weaker neural responses [19]. The composition of Mooney stimuli mainly comprises black and white parts that create a 3D shape of a face [26]. As these stimuli did not contain typical first-order face features (e.g., eyes or nose), they may also trigger a weaker or less consistent N170 response [61]. Latinus and Taylor (2005) found that Mooney stimuli elicited a delayed and enhanced N170 component, but only after participants received a training. We also did not find a face inversion for the Mooney images, but this may reflect that our contrast included only those stimuli that were identified as face and the N170 is associated with a general face detection mechanism [4].

Gamma-band activity has been associated with perceptual coherence. In line with the presented behavioral and ERP results, GBA responses were similar for adolescents with ASD and controls over the first 50 to 200 ms. Our groups did differ in later gamma-band activity from 200 to 350 ms, which has been associated with perceptual binding [39].

Besides the association with higher cognitive functions such as memory and attention [43, 45], early GBA has also been associated with the match of bottom-up and top-down information [43]. Within the MUM model, early GBA reflects the matching of bottom-up signals with memory contents and is enhanced when the matching process yields a positive result [62]. Later bursts have been associated with readout processes like action planning, behavioral control, or memory storage [63]. The prolonged GBA for participants with ASD across time might indicate continued activation of the matching processes.

Another explanation of the prolonged activity in ASD could be an imbalance in precision of top-down predictions and bottom-up sensory processing as suggested by the predictive coding framework [64]. Based on Bayesian decision theory, the framework suggests that we perceive our environment by consistently creating inferences. One part of the inference process is prior knowledge which is extracted from earlier sensory events [65]. These priors are consistently updated when presented with sensory evidence (e.g., Mooney stimulus) and these updates are indexed by prediction errors [66]. Individuals with ASD might have hypo-priors, meaning that whenever they saw a Mooney stimulus their system made larger prediction errors [64]. Cortical responses are considered as an index of prediction errors [65]. The prolonged GBA for individuals with ASD across face and no face responses compared to controls might indicate stronger priors in the controls and prolonged updates within the neural network due to larger prediction errors in the ASD group [66]. The lack of differences for GBA might suggest similar bottom-up perceptual binding, assuming that posterior gamma may be more reflective of a posterior-ventral network (e.g., including inferior-occipital gyri; [67]). Additionally, it might indicate an over-reliance on top-down knowledge and less deviation in perceptual areas [8]. The displayed activation patterns of anterior and posterior clusters across time contribute to the idea of different network activations. The significant decrease of GBA levels from the first to the second time window may suggest that perceptual processes are predominant during early perception, whereas networks in anterior areas are constantly active as part of monitoring and decision-making.

### Limitations

Due to too few trials, we were unable to analyze the ERP contrasts involving the no face responses for the upright stimuli. Therefore, an enhanced understanding of holistic processing in ASD could be accomplished by a different attentional task. For example, Castelhano et al. (2013) used different perceptual states and presentation angles for the same physical stimulus or Sun et al. (2012) scrambled the Mooney stimuli to make them even less “face-like” [19, 29]. To further delineate effects of face processing from object processing, another option would be to employ Mooney stimuli that are objects or noise as contrasts.

We did not find a face inversion effect in our ERP data. While Mooney stimuli do resemble faces, they are only face-like. The inversion effect for realistic faces not only reflects both a decrement in performance when inverted, but also the efficiency and reliability of processing when upright. Thus, it may be that processing a Mooney stimulus upright compared to inverted (at this age) may require a more similar activation pattern that results in non-significant differences in scalp ERP amplitude and latency.

Adolescent development reflected a research area of less focused attention, given the inclusion of adolescents either in child samples or in adult samples, and a period wherein some qualitative aspects of the face processing system are mature, although potentially quantitatively different. Our results suggest an intact face inversion effect for adolescents with ASD and minor quantitative differences on the neural level.

Longitudinal study designs might be most suitable to detect behavioral changes as well as the time course of P1, N170, and gamma-band abnormalities as Webb, Neuhaus, and Faja [68] have suggested significant improvement and “normalization” of face neural circuitry into adolescence and adulthood in ASD, particularly in relation to first-order processing.

The analysis of binding processes could also be further addressed with phase information and cross-frequency coupling [57, 69, 70]. Understanding connectivity in long-range connections and between sensory areas and attentional systems would be important in understanding how top-down processes related to the task directions influence perceptual responses.

## Conclusions

In this paper, we examined behavioral performance, P1, N170, and gamma-band activity in adolescents with ASD and controls during face perception with a carefully selected (IQ-, sex-, and age-matched), narrow range sample. Processing differences may be due to less efficient holistic face processing in ASD, which is required to perceive Mooney stimuli as faces. However, the general similarities between groups suggest that these neural systems are available in individuals with ASD but may be less pronounced or consistently activated. Thus, the fundamental idea of individuals with ASD having an impaired holistic face processing system should be reviewed.

## Additional file

Additional file 1: Geodesic Sensor Net–128 Channel V 2.0. (PDF 746 kb)

## Abbreviations

ADI: Autism Diagnostic Interview
ADOS: Autism Diagnostic Observation Schedule
ASD: Autism spectrum disorder
EEG: Electroencephalography
ERP: Event-related potential
ICA: Independent component analysis
ITI: Inter-trial interval
WISC: Wechsler Intelligence Scale
