# Supplementary figures and images for: Neurophysiological correlates of holistic face processing in adolescents with and without autism spectrum disorder
Source: J Neurodev Disord. 2018 Aug 30;10:27. doi: 10.1186/s11689-018-9244-y (PMC6118009; doi:10.1186/s11689-018-9244-y)

*Geodesic Sensor Net – 128 Channel V 2.0-*

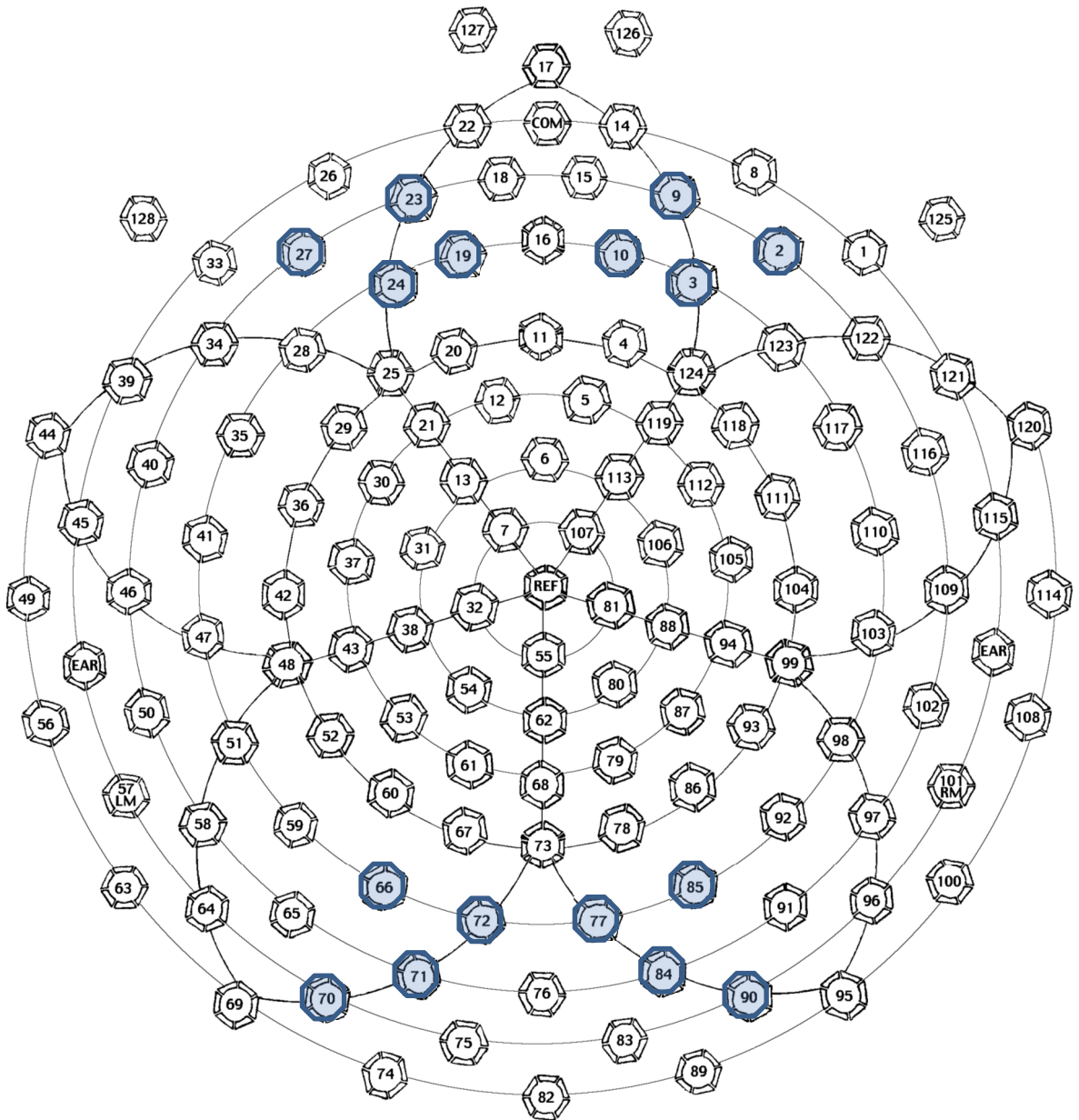

Supplement: Supplementary file 1 — Geodesic Sensor Net–128 Channel V 2.0. (PDF 746 kb) [file 11689_2018_9244_MOESM1_ESM.pdf]
